# Supplementary material for: MetaRibo-Seq measures translation in microbiomes
Source: Nat Commun. 2020 Jun 29;11:3268. doi: 10.1038/s41467-020-17081-z (PMC7324362; doi:10.1038/s41467-020-17081-z)
Supplement: Supplementary file 10 — Supplementary Data 7 [file 41467_2020_17081_MOESM10_ESM.zip › File2/Confidence_High_Taxonomy/386484_out.krona.html]

Javascript must be enabled to view this page.

members
magnitude
magnitudeUnassigned
count
unassigned
taxon
rank

386484\_out

61

2
superkingdom
61

1239
phylum
61

186801
class
61

186802
61
order

186803
family
61

841
61
genus


SRS011084\_contig\_number\_33853SRS013158\_contig\_number\_1669SRS013940\_contig\_number\_12956SRS013951\_contig\_number\_3633SRS014287\_contig\_number\_9802SRS014613\_contig\_number\_20926SRS014855\_contig\_number\_1194SRS014923\_contig\_number\_15806SRS015264\_contig\_number\_18879SRS015578\_contig\_number\_33292SRS015579\_contig\_number\_22265SRS015854\_contig\_number\_8982SRS015890\_contig\_number\_6909SRS015960\_contig\_number\_17248SRS016335\_contig\_number\_36380SRS018623\_contig\_number\_19449SRS018817\_contig\_number\_15140SRS018984\_contig\_number\_10689SRS019068\_contig\_number\_1339SRS019397\_contig\_number\_9540SRS022137\_contig\_number\_11140SRS022609\_contig\_number\_13096SRS024435\_contig\_number\_40532SRS024549\_contig\_number\_4800SRS043001\_contig\_number\_11337SRS045739\_contig\_number\_6908SRS050925\_contig\_number\_29933SRS052027\_contig\_number\_15831SRS053335\_contig\_number\_contig-100\_535.114706SRS054956\_contig\_number\_355SRS055017\_contig\_number\_29429SRS056273\_contig\_number\_26315SRS056519\_contig\_number\_10486SRS058070\_contig\_number\_25185SRS063518\_contig\_number\_20015SRS075341\_contig\_number\_20234SRS076929\_contig\_number\_contig-100\_287.121362SRS077502\_contig\_number\_7878SRS077641\_contig\_number\_13423SRS098061\_contig\_number\_8661SRS098827\_contig\_number\_9735SRS104084\_contig\_number\_contig-100\_907.80476SRS1041130\_contig\_number\_2035SRS104485\_contig\_number\_4282SRS104975\_contig\_number\_20339SRS105153\_contig\_number\_34624SRS1055034\_contig\_number\_9606SRS142503\_contig\_number\_49957SRS142781\_contig\_number\_9921SRS143070\_contig\_number\_13041SRS143722\_contig\_number\_15905SRS143780\_contig\_number\_20031SRS144362\_contig\_number\_26728SRS144714\_contig\_number\_24063SRS147919\_contig\_number\_20645SRS148091\_contig\_number\_10442SRS148159\_contig\_number\_16725SRS149181\_contig\_number\_30103SRS149879\_contig\_number\_24008SRS893378\_contig\_number\_20941
166486
60
species

species
1

SRS077454\_contig\_number\_12685
2049040
